# Indole Alkaloids from *Catharanthus roseus*: Bioproduction and Their Effect on Human Health

**DOI:** 10.3390/molecules20022973

**Published:** 2015-02-12

**Authors:** Lorena Almagro, Francisco Fernández-Pérez, Maria Angeles Pedreño

**Affiliations:** Department of Plant Biology, Faculty of Biology, University of Murcia, Murcia 30100, Spain; E-Mails: lorena.almagro@um.es (L.A.); f.fernandezperez@um.es (F.F.-P.)

**Keywords:** bioactivity, bioproduction, *Catharanthus roseus*, indole alkaloids

## Abstract

*Catharanthus roseus* is a medicinal plant belonging to the family Apocynaceae which produces terpenoid indole alkaloids (TIAs) of high medicinal importance. Indeed, a number of activities like antidiabetic, bactericide and antihypertensive are linked to *C. roseus*. Nevertheless, the high added value of this plant is based on its enormous pharmaceutical interest, producing more than 130 TIAs, some of which exhibit strong pharmacological activities. The most striking biological activity investigated has been the antitumour effect of dimeric alkaloids such as anhydrovinblastine, vinblastine and vincristine which are already in pre-, clinical or in use. The great pharmacological importance of these indole alkaloids, contrasts with the small amounts of them found in this plant, making their extraction a very expensive process. To overcome this problem, researches have looked for alternative sources and strategies to produce them in higher amounts. In this sense, intensive research on the biosynthesis of TIAs and the regulation of their pathways has been developed with the aim to increase by biotechnological approaches, the production of these high added value compounds. This review is focused on the different strategies which improve TIA production, and in the analysis of the beneficial effects that these compounds exert on human health.

## 1. Introduction

*Catharanthus* is a perennial tropical medicinal plant belonging to the Family Apocynaceae which comprises eight species, seven endemic to Madagascar (*C. coriaceus*, *C. lanceus*, *C. longifolius*, *C. ovalis*, *C. roseus*, *C. scitulus*, *C. trichophyllus*), and one, *C. pusillus*, from India. Specifically, *C. roseus* is a decorative and curing plant of enormous pharmaceutical interest because it is nothing less than a chemical factory, producing more than 130 different terpenoid indole alkaloids (TIAs), some of which exhibit strong and important pharmacological activities [1]. In fact, vinblastine and vincristine are commercial TIAs used in anticancer chemotherapy, and together with a number of related semi-synthetic compounds, are collectively named the Vinca alkaloids. Currently, vinblastine, vincristine, vinorelbine and vindesine have been used in clinical trials, although only vinblastine, vincristine and vinorelbine have been approved for medical treatment in the United States [2]. Vinflunine, a fluorinated analogue of vinorelbine, has been approved in Europe [3,4]. Vincristine and vinblastine also show a strong antimicrobial activity [5]. In addition, *C. roseus* also produces ajmalicine and serpentine, which are monoterpenic indole alkaloids used as anti-hypertensive and anti-neuro-inflammatory agents, yohimbine which is mainly used in treatments for erectile dysfunction, and vindolicine used for the development of antidiabetic therapeutics. 

The large interest in the anticancer compounds vinblastine and vincristine, which derive from the coupling of catharanthine and vindoline, contrasts with the low amounts of these compounds found in the plants, making their extraction a very expensive process. These low levels are mainly associated to the spatial separation of biosynthetic sites where these compounds are produced in the plant and to the high degree of specialization of some leaf cells where the assembly of specific steps of the TIA biosynthetic pathway occurs [6]. In fact, catharanthine is accumulated almost exclusively in the wax exudates on the leaf surface, whereas vindoline is produced in specialized internal leaf cells, suggesting that an involvement of transport processes are needed for their coupling to take place [7]. Recently, an ABC transporter, CrTPT2, whose primary function consists in enhancing the transport and hence, the accumulation of catharanthine in the leaf epidermal surface, has been identified [6]. However, the physical separation of catharanthine and vindoline observed by Yu and DeLuca [6] is probably a limiting factor in very young leaves, where AVLB was actually shown to be absent [8], but definitely not in developed leaves, where the dimer AVLB was repeatedly reported to be abundant [8,9,10,11,12]. In fact, Carqueijeiro *et al.* [12] demonstrated that catharanthine, vindoline and AVLB were accumulated in the vacuoles of mesophyll cells by a specific proton antiport system, dependent on the transtonoplast pH gradient generated by V-H^+^-ATPase and V-H^+^-PPase using vacuoles isolated from leaves of adult plants. 

In addition, researchers have looked for alternative sources and strategies to produce TIAs in high amounts. In fact, the low levels of the TIAs with anticancer activity found in plants have stimulated an intense research effort aiming to obtain *in vitro* *C. roseus* cultures with a higher production of these TIAs. Technologically, Zhao and Verpoorte showed that although *C. roseus* cells can be cultivated in bioreactors, the TIA biosynthesis is extremely low, which prevents their industrial production. To increase this production, several approaches were tried [13] using *C. roseus* cell cultures, being genetic modification or metabolic engineering the most promising biotechnological alternatives for producing these compounds [1]. 

After providing an overview of pharmacological activities of some TIAs and semi-synthetic Vinca alkaloids, this review aims to summarize and highlight the key issues of TIA-related research in the 21st century, with particular emphasis on the empirical strategies developed for improving TIA production using plants and *in vitro* cultures of shoots, hairy roots and cells. Special attention is also focused on rational approaches, which are the most promising strategies to improve TIA production in the future.

## 2. Pharmacological Activities

### 2.1. Antiviral and Antimicrobial Activity

Microbial resistance has become an increasing problem for human health, and therefore, several researchers have focused on the discovery of new antimicrobial agents. In this sense, numerous studies have been made on antimicrobial activity of natural products, including alkaloids [14]. In fact, ethanolic extracts from different parts of *C. roseus* like leaves, stems, roots and flowers have been tested for antibacterial activity. The leaf extracts showed significantly higher activity, suggesting that bioactive compounds of *C. roseus* can be potentially exploited as antibacterial agents [15]. However, there are few studies that demonstrate the antiviral and antimicrobial activities of a single alkaloid obtained from *C. roseus*. Özçelik *et al.* [16] showed the antiviral effect of yohimbine, found naturally in *Pausinystalia yohimbine* [17] and in *C. roseus*, on herpes simplex virus (type 1) with a cytopathogenic effect at 0.8 µg/mL. In addition, yohimbine exhibited antifungal activity against the opportunistic pathogen *Candida albicans*, a causal agent of opportunistic oral and genital infections in humans [18]. In the same way, catharoseumine, a monoterpenic indole alkaloid isolated from the whole plant of *C. roseus* possessing a unique peroxy bridge, was identified as a potential inhibitor against protozoan parasite falcipain-2 (that causes malaria) showing an IC_50_ value of 4.06 µM [19]. Similarly, vinblastine and vincristine showed antiparasitic effects against *Trypanosoma cruzi* that causes trypanosomiasis in humans, inhibiting its mitosis and affecting its cell shape in a dose-dependent manner [5]. Indeed, the use of 15 µM vinblastine and 50 µM vincristine inhibited both nuclear division and cytokinesis, and affected cell shape, while the joint effect of 3 µM vinblastine and 10 µM vincristine inhibited cytokinesis without affecting cell cycle progression. 

### 2.2. Antidiabetic and Antioxidant Properties

Diabetes mellitus is considered one of the most important causes of mortality and to date, there is not a completely effective treatment for its healing [20,21,22]. Due to the side effects of insulin and oral hypoglycemic agents, currently bioactive plant natural metabolites which have antidiabetic activity are a promising alternative. In this way, Tiong *et al.* [23] examined the hypoglycemic and antioxidant activity of vindoline, vindolidine, vindolicine and vindolinine obtained from *C. roseus* (L.) G. Don leaves. These authors indicated that these compounds provoked a rise of glucose absorption in pancreatic or myoblast cells, being vindolicine the compound that showed the highest activity. In addition, vindolidine, vindolicine and vindolinine had a high inhibitory activity against protein tyrosine phosphatase-1B inhibitory activity, indicating that these compounds could be used for diabetes. Vindolicine also exhibited the highest antioxidant effects in both the oxygen radical absorbance capacity and 1,1-diphenyl-2-picrylhydrazyl tests, and this compound also decreased H_2_O_2_-induced oxidative damage to pancreatic cells. Therefore, in the future, vindolicine could be used as antidiabetic agent.

### 2.3. Potential Effects of Yohimbine on Erectile Dysfunction

Up until the late 1990s, yohimbine, one α-2 adrenergic antagonist, was one of the few oral pharmacological agents prescribed for the treatment of erectile dysfunction. However, relatively few well-designed studies on the therapeutic effect of yohimbine have been completed [24]. In general, the treatment with yohimbine alone presents low efficacy in patients with erectile dysfunction, while the combined therapy of yohimbine and L-arginine (that enhances the nitric oxide pathway) is more effective to improve the erectile function [25]. 

### 2.4. Potential Effects of Ajmalicine and Serpentine as Anti-Neuro-Inflammatory Agents

Inflammation of the central nervous system or neuro-inflammation is the most important feature of neurodegenerative diseases such as Alzheimer’s [26,27]. In this sense, irregular action of the central nervous system triggers an increase in cyclooxygenase-II levels, which provokes neuroinflammation [28]. Cyclooxygenases catalyse an early step in the biosynthesis of prostanoids, which potentiate the inflammatory cascade causing neuronal damage. Manigandan *et al.* [29] evaluated the inhibitory effect of ajmalicine, vindoline, catharanthine, serpentine and tabersonine on cyclooxygenase-II, observing that particularly serpentine acted as a potent anti-neuroinflammatory agent since it inhibited the receptor of cyclooxygenase-II. 

### 2.5. Potential Effects of Ajmalicine on Vascular Disorders

Adrenergic receptor antagonists have been widely used for the treatment of hypertension. In this sense, ajmalicine has been used as a hypotensive agent since it acted as an α-adrenergic receptor antagonist [30]. Similarly, serpentine is also considered a hypotensive agent [31]. 

### 2.6. Antitumour Properties

The Vinca alkaloids have generally been used in the treatment of cancer [2,32]. These compounds repress cell growth because they alter the microtubular dynamics, and ultimately this provokes apoptosis. Semi-synthetic compounds similar to vinblastine and vincristine have been developed to increase their therapeutic action [33].

Vinblastine is used in particular for the treatment of Hodgkin’s disease, besides lymphosarcoma, choriocarcinoma, neuroblastoma, carcinoma of breast and lung, and lymphocytic leukemia [34,35]. Anhydrovinblastine, the direct precursor of vinblastine, also showed significant *in vitro* cytotoxic effect against human non-small cell lung cancer C4 and human cervical carcinoma, human leukemic cells, and A431 human carcinoma cells [36]. 

Vincristine is an oxidised form of vinblastine that arrests mitosis in metaphase and is very effective for treating acute lymphoblastic leukaemia in both children and adults. It is also used against Hodgkin’s disease, Wilkins’s tumour, neuroblastoma, and reticulum cell sarcoma [2,32]. In addition, vincristine has also been used in the treatment of multiples non-malignant hematologic disorders like autoimmune and thrombotic thrombocytopenia, and hemolytic uremic syndrome [2,32]. 

On the other hand, the cytotoxic effect of catharoseumine, which is a monoterpenic indole alkaloid isolated from the whole plant of *C. roseus*, was tested in different human tumour cell lines showing only a moderate cytotoxic effect against HL-60 cell line [19].

#### 2.6.1. Semi-Synthetic Derivatives of Dimeric Alkaloids

Vinblastine and vincristine are present at low concentration levels in *C. roseus* plants, however their production is more than enough to generate derivative compounds with improved pharmacological properties. Vindesine is a semi-synthetic derivative from vinblastine, which provokes the arrest of cells in metaphase since vindesine inhibits the function of tubulin. This drug is used to treat diseases such as acute leukaemia, malignant lymphoma, Hodgkin's disease, acute erythraemia and acute panmyelosis. Nowadays, this compound is being examined for its potential to synergize with interferon, and for its value as therapeutic in preventing metastasis [37]. Vindesine was also shown to be effective in combined therapies of soft tissue sarcomas. In fact, Rhomberg *et al.* [38] analyzed the combination of razoxane, vindesine and radiotherapy on the dynamics of metastasis in advanced soft tissue sarcomas. The combination inhibited the development of metastasis in a majority of the patients and prolonged their survival.

Vinorelbine is other semi-synthetic derivative obtained from vinblastine, although it is less neurotoxic than its precursor. This compound showed a high antitumour effect on patients with breast or prostate cancer because vinorelbine inhibits mitosis through its interaction with tubulin. In addition, vinorelbine has been approved by Food and Drug Administration to treat patients with advanced lung cancer in the United States [39]. Because of this efficacy and its good safety, vinorelbine has been administered in combination with other agents (capecitabine, epirubicin, docetaxel *etc.*..) for the treatment of different types of cancers [40]. 

Vinflunine is the first fluorinated microtubule inhibitor synthesised from vinorelbine. Vinflunine is different from others Vinca alkaloids because this compound binds weakly to tubuline, showing an improved tolerance profile as a result of its less neurotoxicity. Three possible effects of vinflunine have been described: its action against tubulin and microtubules, its capacity to disrupt newly formed blood vessels, and its ability to reduce the metastatic process. These findings support the hypothesis that vinflunine might have not only a tumour-cytostatic effect but also a specific antiangiogenic effect. Clinically, vinflunine is being seen for its potential role for treating of mammary carcinoma and non-small cell lung cancer, amongst others [4,41].

#### 2.6.2. Mechanism of Action of Dimeric Alkaloids

Microtubules (MTs) are components of the cytoskeleton and play very important role in a great numbers of cellular processes. They are involved in chromosome separation during mitosis and meiosis, and are the major constituents of mitotic spindles, besides they are involved in maintaining cell structure, transport and many others cell functions. MTs are synthesized from α,β-tubulin heterodimers which polymerize end-to-end into linear protofilaments. These structures are, in a dynamic polymerization and depolymerization, at their ends. The assembly and disassembly of the MT polymers are regulated by the binding of tubulin and guanosine 5-triphosphate. Any interference with this MT dynamics can provoke a cell cycle arrest and lead to programmed cell death or apoptosis [42].

MT dynamics, and therefore cell division, can also be perturbed by small molecules, which are usually divided into two groups: (i) MT-stabilizing agents that prevent then depolymerization; and (ii) MT-depolymerizing agents that inhibit their formation. Vinca alkaloids fall inside the second group, as they arrest tumour cells during mitosis by binding at the surface between two tubulin heterodimers next to the exchangeable guanosine 5-triphosphate-binding site [43] and depolymerizing the MTs. This leads to cell cycle arrest in mitosis [44,45]. At high concentration Vinca alkaloids lead to the formation of large tubulin polymers called “Paracristal” and as a result, the tumoral cells are stopped in mitosis and immediately they die [46]. However, when the levels of Vinca alkaloids are low, the cells are arrested in mitosis and the cells die after a long time of incubation. 

**Figure 1:**
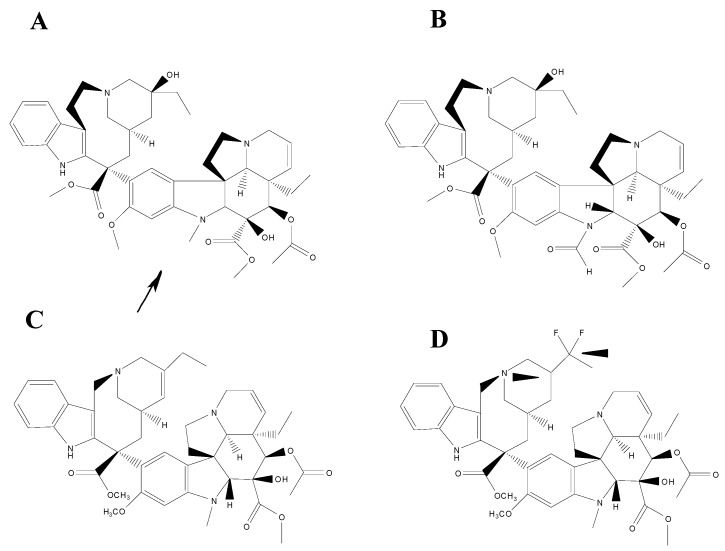
Chemical structures of the four *Vinca* alkaloids vincristine (**A**), vinblastine (**B**), vinorelbine (**C)** and vinflunine (**D**). The arrow indicates the principal difference between vincristine (A) and vinblastine (B). The arrow heads indicate the principal difference between vinorelbine (C) and vinflunine (D).

Since the binding of natural Vinca alkaloids, vinblastine and vincristine, as well as their semi-synthetic analogues, vinorelbine and vinflunine, is linked to tubulin self-association, their affinities can be determined and their binding are entropically driven so that the overall affinities decrease in the following order: vincristine > vinblastine > vinorelbine > vinflunine (Figure 1). 

The affinity of the four Vinca alkaloids for tubulin heterodimers appears to be almost identical however [47], the major differences amongst them are due to the distinct affinities of the resulting ligand heterodimers for polymerized spirals. All of Vinca alkaloids, in their respective complexes with α,β-tubulin, exhibit overall similar van der Waals forces and electrostatic interaction energy terms thereby defining a common binding site. However, the vindoline domain is used to bind tubulin heterodimers, while the catharanthine domain provides a cytotoxic effect [48]. Their binding site to MTs is lined by the side chains of a number of mostly non-polar amino acids from both tubulin monomers. The formyl group on the indole ring of vincristine that replaces the methyl in vinblastine is relevant for different spectra of pharmacological activity and dose-related toxicities, as well as differential cellular uptake and retention characteristics. On the other hand, the saturated double bond and the two fluorine atoms that differentiate vinflunine from vinorelbine provide only a marginally improved electrostatic interaction [49]. Even though their interaction with tubulin has been characterized, additional studies have revealed new mechanisms of action, such as their interplay with MT associated protein, inhibition of amino acid metabolism and interaction with calmodulin. This interaction with calmodulin could explain the differences in efficacy of vinflunine that, in spite of its binds to tubulin is lower than vinblastine or vincristine, displays a better activity than vinblastine against murine tumours and human tumour xenografts. Vinflunine turned out to be a better inhibitor binding to calmodulin, therefore presents higher efficiency and lower toxicity *in vivo* [50]. A recent study based on vinorelbine supports the hypothesis about the importance of impact of “secondary” targets in overall action of Vinca alkaloids [51]. This could explain why, despite of very similar binding parameters on tubulin, vinorelbine and vinblastine had such a different use in clinics [52].

## 3. Keynotes Based on the Use of *C. roseus* Plants as Biofactories for Enhancing the Production of Terpenoid Indole Alkaloids

As stated above, the production of TIAs by *C. roseus in vitro* cultures is a feasible technology pursued by industrial and academic interests. It is crucial to take into account, prior to the use of high producer *in vitro* cultures of TIAs, the right choice of the *C. roseus* cultivar which provides the best TIA levels. Although different studies based on HPLC metabolic profiling showed nearly equal amounts of TIAs in different cultivars of *C. roseus*, some *C. roseus* cell lines produced high levels of TIAs. In fact, the selection and development of a cell line which synthesizes and accumulates TIAs depends on critical steps like the selection of appropriated explants (type, size, age, amongst others*.*) which are used for initiating the cell culture as well as culture conditions (nutrients, growth regulators, pH, temperature, oxygen and light, amongst others), which in turn, are critical factors on which the success of *in vitro* cultures depends. In addition, screening of cell lines along successive subcultures [53,54,55] has also enabled the selection of high-yielding cell lines although they are usually unstable and tend to decrease their production after periodical subcultures. Mannonen *et al.* [56] used different cryopreservation methods, several cryoprotectants, and cooling and thawing processes. The use of mineral oil has been a useful strategy for preservating cells which restarted their growth in liquid fresh culture media [57]. 

On the other hand, several studies carried out using whole plants, shoot, callus and cell cultures of *C. roseus* demonstrated that TIA production is dependent on both cell differentiation and organogenesis [58]. Endo *et al.* [59] observed that the pattern of TIA production in root and shoot cultures of *C. roseus* was similar to that found in roots and leaves of whole plants. However, many TIAs cannot be synthesized by *C. roseus* cell cultures since the activation of biosynthetic pathways is tissue-specific and developmentally regulated. Shukla *et al.* [60] observed that levels of vindoline and catharanthine enhanced in *C. roseus in vitro* cultures as the level of cell differentiation increased. However, these compounds were only detected in elicited conditions, the highest amounts of catharanthine and vindoline occurring in shoots (0.0039% and 0.0013% on a dry weight (DW) basis, respectively) compared with the levels found in callus (0.00019% and 0.00015% on a DW basis, respectively). The culture of hairy roots is another strategy for TIA production, since roots grow rapidly in hormone-free liquid medium, and they are composed of differentiated cells genetically stable, able to produce a broad spectrum of TIAs. In fact, the production of ajmalicine, serpentine, and catharanthine has been repeatedly reported by different authors, sometimes reaching levels superior to non-transformed root cultures (around 0.2%–0.4% DW) [61,62]. Production of vindoline has also been detected in hairy roots, at very low levels (0.04%–0.08% DW) [61,62,63]. 

## 4. Empirical Strategies for Improving TIA Production

### 4.1. Empirical Optimization of the Culture Medium and Culture Conditions

The composition of the culture medium as well as the physical environmental conditions, and the addition of specific compounds to the culture medium have been proved to increase TIA yields in *C. roseus in vitro* cultures, sometimes in a dramatic way. Thus, reducing or removing nitrate and/or phosphate ions from the medium leads to a significant decrease of cell growth but remarkably, an increase in TIA accumulation [64]. On the other hand, the addition of KCl (4 g/L) did not inhibit cell growth and increased threefold the production of ajmalicine and serpentine in comparison to the levels found in control compact callus clusters cultures of *C. roseus* [65] (Table 1). Similarly, the addition of NaCl (1.7 g/L) caused a rise in catharanthine levels (around 90%), while the same KCl concentration resulted in an increase of catharanthine around 300%. Moreover, the effect of a higher NaCl concentration (2.9 g/L) increased the content of vindoline, catharanthine, vinblastine and vincristine in *C. roseus* seedlings [66]. The highest levels of TIAs were obtained by adding 250 mM mannitol (four- to five-fold higher in comparison to the controls) to compact callus clusters cultures of *C. roseus* [65] (Table 1). The carbon source concentration is another limiting factor for the TIA biosynthesis. In fact, Schlatmann *et al.* [67] observed that the presence of a low glucose concentration correlated with a high ajmalicine production level. Zhao *et al.* [68] observed an increase of ajmalicine, serpentine and catharanthine (a total alkaloid yield of over 43 mg/L), when the concentration of sucrose increased in the culture medium of compact callus clusters of *C. roseus* (Table 1). Similarly, Jung *et al.* [69] observed an increase in the levels of catharanthine when the carbon source changed (fructose for sucrose) in *C. roseus* hairy root cultures.

Several authors have showed that the production of ajmalicine was significantly lower when a low *C. roseus* cell *inoculum* density was used although catharanthine levels were nearly the same at high and low cell densities [70,71].

The immobilization of plant cells provides resistance to the diffusion and it could increase the levels of TIAs, specially using a high cell density. In fact, several carriers have been used to immobilize cells of *C. roseus* [1]. In this sense, an increase of ajmalicine levels and its excretion to the medium when *C. roseus* cells were immobilized with calcium alginate was observed by Asada and Shuler [72]. Also, Lee and Shuler [71] observed that high inoculum density in *C. roseus* cultures in which cells are immobilized on alginate beads produced a high ajmalicine concentration (120 mg/L) [71] (Table 1). 

**Table 1:** Strategies to increase the production of TIAs in *C. roseus*.

Material	Strategy	Total TIAs	Observations	Refs
Compact callus cluster	Medium supplemented with mannitol (250 mM)	4.13-fold increase (75 mg/L)	5.05-fold increase in catharanthine (19.7 mg/L)	[65]
			2.77-fold increase in serpentine (13.6 mg/L)	
			4.54-fold increase in ajmalicine (42.3 mg/L)	
	Medium supplemented with KCl (4g/L)	3.15-fold increase (57.8 mg/L)	3.15-fold increase in catharanthine (12.3 mg/L)	
			2.42-fold increase in serpentine (11.9 mg/L)	
			3.62-fold increase in ajmalicine (33.5 mg/L)	
Compact callus cluster	Medium supplemented with sucrose (50 g/L)	1.16-fold increase (46.7 mg/L)	1.25-fold increase in serpentine (10 mg/L)	[68]
			1.20-fold increase in ajmalicine (14.5 mg/L)	
Suspension cultured cells	High cell density (200 g FW/L)	ND	120-fold increase in ajmalicine (60 mg/L)	[73]
Immobilized cells	High cell density (100 g FW/L)	ND	2-fold increase in ajmalicine (120 mg/L)	[71]
Immobilized cells	Elicitation with *Phytophthora* *cactorum*	ND	45-fold increase in ajmalicine (90 mg/L)	[72]
Shoot culture	MS medium supplemented with plant growth regulators	ND	The concentrations of 8.90 µM BA and 2.85 µM IAA increased the production of ajmalicine (0.85 g/L)	[74]
Callus	MS medium supplemented with plant growth regulators	ND	The concentrations of 2.21 µM BA and 5.7 µM IAA increased the production of catharanthine (0.12 mg/g DW)	[68]
			ajmalicine (0.35 mg/g DW)	
			vindoline (0.19 mg/g DW)	
			serpentine (0.53 mg/g DW)	
Immobilized cells	Variation of O_2_ and CO_2_ concentration	ND	1.1-fold increase in ajmalicine (275 g/L )	[75]
Suspension cultured cells	Feeding with loganin and triptamine	ND	17.73-fold increase in strictosidine (53.19 µmo/g DW)	[76]
			6.4-fold increase in ajmalicine (3.2 µmol/g DW)	
Compact callus cluster	Feeding with succinic acid (10 mM)	4.86-fold increase (73 mg/L)	3.5-fold increase in catharanthine (7 mg/L)	[65]
			7.5-fold increase in serpentine (15 mg/L)	
			16-fold increase in ajmalicine (32 mg/L)	
	Feeding with tryptamine (3.12 mM)	3.86-fold increase (58 mg/L)	2.5-fold increase in catharanthine (5 mg/L)	
			7-fold increase in serpentine (14 mg/L)	
			15.5-fold increase in ajmalicine (31 mg/L)	
	Feeding with tryptophan (2.44 mM)	5.53-fold increase (68 mg/L)	2.5-fold increase in catharanthine (5 mg/L)	
			6-fold increase in serpentine (12 mg/L)	
			14-fold increase in ajmalicine (28 mg/L)	
Hairy root culture	Feeding with geraniol (0.5 mM)	ND	1.5-fold increase in tabersonine (1.4 mg/g DW)	[77]
Hairy root culture	Elicitation with sodium nitroprusside (0.1 mM)	1.42-fold increase (3.7 mg/g DW)	2-fold increase in lochnericine (1 mg/g DW)	[78]
			2.3-fold increase in tabersonine (0.7 mg/g DW)	
			2-fold increase in ajmalicine (0.7 mg/g DW)	
Suspension cultured cells	Elicitation with MeJA (101.9 µM)	1.33-fold increase (2.2 mg/g DW)	2-fold increase in tabersonine (3.8 mg/g DW)	[79]
Suspension cultured cells	Elicitation with MeJA (100 µM)	ND	27.44-fold increase in ajmalicine (137.2 mg/L)	[80]
			11.12-fold increase in catharanthine (55.6 mg/L)	
Hairy root culture	Elicitation with MeJA (250 µM)	1.32-fold increase (49 mg/L)	7-fold increase in ajmalicine (6.34 mg/g DW)	[81]
			2.9-fold increase in serpentine (1.71 mg/g DW)	
			3-fold increase in ajmaline (12 mg/g DW)	
			3-fold increase in catharanthine (4.34 mg/g DW)	
Suspension cultured cells	Elicitation with *Trichoderma viride*	ND	7.9-fold increase in ajmalicine (0.166 mg/g DW)	[82]
Suspension cultured cells	Elicitation with the protein of *Phytophthora boehmeriae* (BP90)	ND	4-fold increase in catharanthine (20 mg/L)	[83]
Suspension cultured cells	Elicitation with CDs	ND	40-fold increase in ajmalicine (200 mg/L)	[80]
			17-fold increase in catharanthine (85 mg/L)	
Suspension cultured cells	Elicitation with UV-B light	ND	3-fold increase in catharanthine (0.12 mg/g DW)	[84]
			117.6-fold increase in vindoline (0.06 mg/g DW)	
Suspension cultured cells	Elicitation with UV-C light	ND	18-fold increase in ajmalicine (90 mg/L)	[73]
			10-fold increase in catharanthine (50 mg/L)	
Plant	Elicitation with chromium (50 µM)	ND	1.5-fold increase in vincristine (2 µg/g DW)	[35]
			2.16-fold increase in vinblastine (2.25 µg/g DW)	
Suspension cultured cells	Elicitation with *Aspergillum niger* mycelium and tetramethyl ammonium bromide	3.84-fold increase (96 mg/L)	21-fold increase in ajmalicine (63 mg/L)	[85]
			17-fold increase in catharanthine (17 mg/L)	
	Elicitation with malate and sodium alginate	3.28-fold increase (82 mg/L)	13.6-fold increase in ajmalicine (41 mg/L)	
			26-fold increase in catharanthine (26 mg/L)	
Suspension cultured cells	Elicitation with MeJA and CDs	ND	90-fold increase in ajmalicine (450 mg/L)	[73]
			31-fold increase in catharanthine (155 mg/L)	
	Elicitation with MeJA, CDs and UV-C light	ND	2.3-fold increase in ajmalicine (1040 mg/L) (85 mg/g DW)	
			1.26-fold increase in catharanthine (196 mg/L) (10 mg/g DW)	
Suspension cultured cells	Overexpression of *STR*	24.6 fold in increase (123 mg/L)	_	[86]
Suspension cultured cells	Overexpression of *TDC* and feeding with loganin and secologanin	125 fold in increase (625 mg/L)	_	[87]
Hairy root culture	Overexpression of *DAT*	ND	4-fold increase in hörhammericine (0.16 mg/g DW)	[88]
Hairy root culture	Overexpression of CrPrx	1.5-fold increase (85 mg/g DW)	5-fold increase in serpentine (3.7 mg/g DW)	[89]
			3-fold increase in ajmalicine (0.35 mg/g DW)	
Hairy root culture	Overexpression of *DXS*	NV	1.66-fold increase in ajmalicine (1.5 mg/g DW)	[90]
			1.66-fold increase in lochnericine (1 mg/g DW)	
	Overexpression of *ASα*	NV	1.25-fold increase in lochnericine (2.5 mg/g DW)	
	Overexpression of *G10H/DXS*	0.0072 mg/g DW	1.35-fold increase in tabersonine (0.9 mg/g DW)	
			1.15-fold increase in lochnericine (1.4 mg/g DW)	
	Overexpression of *ASα/DXS*	0.015 mg/g DW	1.16-fold increase in tabersonine (1.7 mg/g DW)	
			1.18-fold increase in lochnericine (2 mg/g DW)	
Leaves	Transient overexpression of *GPPS*	ND	1.6-fold increase in vindoline (2.5 mg/g DW)	[91]
Tobacco cell cultures	Overexpression of *TDC/STR*	ND	Enhancement in strictosidine (5.3 mg/L)	[92]
*Morinda citrifolia* cell cultures	Overexpression of *TDC/STR*	ND	Enhancement in strictosidine (21.2 mg/L)	
*Cinchona officinalis* hairy root culture	Overexpression of *TDC/STR*	ND	Enhancement in strictosidine (1.95 mg/g FW)	[93]
*Saccharomyces cerevisiae*	Overexpression of *STR/SGD*	ND	Enhancement in strictosidine (2000 mg/L)	[94]
Hairy root culture	Overexpression of transcription factor *CrWRKY1*	ND	3-fold increase in serpentine (0.291 mg/g DW)	[95]
			10-fold increase in ajmalicine (0.015 mg/g DW)	
Leaves	Transient overexpression of transcription factor *CrMPK3*	ND	3.52-fold increase in serpentine (0.061 mg/g DW)	[96]
			2.66-fold increase in vindoline (4.1 mg/g DW)	
			1.44-fold increase in catharanthine (1.3 mg/g DW)	
			2-fold increase in vincristine (1.75 mg/g DW)	
Hairy root culture	Overexpression of transcription factor *ORCA3*	ND	2.5-fold increase in catharanthine (5.6 mg/g DW)	[97]
Hairy root culture	Overexpression of transcription factor *ORCA2*	ND	2-fold increase in catharanthine (4.8 mg/g DW)	[98]
Transgenic plant	Overexpression of *ORCA3* and *G10H*	ND	3.03-fold increase in vindoline (2.1 mg/g DW)	[99]
			2.29-fold increase in catharanthine (4.6 mg/g DW)	
			6.30-fold increase in ajmalicine (0.315 mg/g DW)	
			1.08-fold increase in anhydrovinblastine (10.2 mg/g DW)	
			10.2-fold increase in vinblastine (0.27 mg/g DW)	

Abbreviations: DW, dry weight; FW, fresh weight; ND, not determined; NV, no variation; TIAs, Terpenoid indole alkaloids; STR, strictosidine synthase; TDC, tryptophan decarboxylase; DAT, deacetylvindoline 4-O-acetyltransferase; CrPrx, *C. roseus* peroxidase; DXS, 1-deoxy-D-xylulose-synthase; G10H, geraniol-10-hydroxylase; ASα, anthranilate synthase α subunit; GPPS, Geranyl diphosphate synthase; CrMPK3, *C. roseus* mitogen activated protein kinase 3, MeJA, methyl jasmonate; CDs, cyclodextrins; BA, benzyl adenine; IAA, indole-3-acetic acid.

As regards the effect of plant growth regulators, 2,4-dichlorophenoxyacetic acid inhibited TIA production [100,101], even when the precursors, loganin and tryptamine, are added to *C. roseus* cell cultures [76]. Similarly, the addition of ethylene inhibited the production of ajmalicine [102]. However, the combination of benzyladenine or kinetin with indole-3-acetic acid led to an enhancement of TIA production (total alkaloid yield of over 45 mg/L). Moreover, Satdive *et al.* [74] found high levels of ajmalicine (over 0.85 g/L) released in the spent media of *C. roseus* shoot grown in the presence of a high level of benzyladenine (8.90 mM) and a low level of indole-3-acetic acid (2.85 mM) (Table 1), whereas the addition of a high concentration of indole-3-acetic acid (11.42 mM) and a low concentration of benzyladenine (2.22 mM) resulted in high levels of ajmalicine (over 0.40 g/L) being accumulated in the shoots. In addition, TIA biosynthesis improved when *C. roseus* callus were treated with benzyladenine and indole-3-acetic acid and exposed to the light, especially vindoline (0.19 mg/g DW) and serpentine (0.53 mg/g DW) compared with callus that was not exposed to light [101].

On the other hand, Lee-Parsons and Shuler [75] showed that different O_2_ concentration decreased both cell growth and ajmalicine biosynthesis in *C. roseus* immobilized cells. However, the ajmalicine biosynthesis was unaffected when a high concentration of O_2_ and CO_2_ was used. 

### 4.2. Feeding with Precursors and Elicitation as Empirical Strategies for Increasing the Production of TIAs

Sometimes, feeding with specific precursors has proved to be a successful strategy to increase the levels of TIAs using *in vitro* cultures. Specifically, tryptophane, tryptamine, geraniol, 10-hydroxygeraniol, loganin and secologanin have been the precursors most extensively added [65,76,77,100,103,104,105]. In fact, the combined use of tryptamine and loganin improved the accumulation of ajmalicine [100] and provoked a high accumulation of strictosidine [76] in *C. roseus* cell cultures. Also, the addition of succinic acid, tryptamine and tryptophan to the culture medium significantly increased ajmalicine and catharanthine levels [65] (Table 1). Similarly, the levels of ajmalicine and strictosidine were enhanced when *C. roseus* cell cultures were fed with loganic acid, loganin and secologanin [105]. More recently, feeding experiments with geraniol (0.5 mM), hydroxygeraniol or loganin provoked a marked increase in the accumulation of tabersonine (around 1.4 mg/g DW) in *C. roseus* hairy root cultures [77] (Table 1), but the most spectacular results were found in loganin feeding experiments where TIA accumulation improved significantly compared to non-transformed cells [104].

On the other hand, elicitation is one of the most effective established strategies to improve secondary metabolite production in plant platforms [106]. Both abiotic and biotic elicitors have been extensively used for improving TIA production in *C. roseus in vitro* cultures since the beginning of the 1990s [105,107,108,109]. In fact, abiotic elicitors like metal ions and UV light, as well as biotic elicitors such as fungal preparations and yeast extracts, hydrolytic enzymes, chemicals (cyclodextrins (CDs) and signalling molecules like nitric oxide, jasmonic acid and derived compounds such as methyl jasmonate (MeJA) and acetylsalicylic acid were commonly used [73,84,85,110,111,112]. In this way, the treatment of *C. roseus* hairy root cultures with 0.1 mM sodium nitroprusside (NO donor) caused a significant increase of serpentine, catharanthine, ajmalicine, hörhammericine, lochnericine and tabersonine [78] (Table 1). Also working with hairy roots of *C. roseus* treated with 101.9 µM of jasmonic acid, Goldhaber-Pasillas *et al.* [79] showed an increase of 190% in tabersonine (Table 1). In the same way, Lee-Parsons *et al.* [110] showed that the addition of 100 µM MeJA to *C. roseus* cell cultures increased the ajmalicine extracellular production (10.2 mg/L) in a dose and time-dependent manner. MeJA also triggered the production of ajmalicine (137.2 mg/L) and catharanthine (55.6 mg/L) in *C. roseus* cell cultures [73]. This elicitor was also effective in accumulating ajmalicine (6.34 mg/g DW) serpentine (1.71 mg/g DW), ajmaline (12 mg/g DW) and catharanthine (4.34 mg/g DW) in *C. roseus* hairy roots elicited with 250 μM MeJA [81] (Table 1). Likewise, the addition of 1 mM of acetylsalicylic acid provoked an increase of 505% total alkaloids in *C. roseus* cells transformed with *Agrobacterium tumefaciens* [113]. 

Several studies carried out with fungal preparations have proved to be a successful approach to increase TIA production in *C. roseus in vitro* cultures. Indeed, a maximum yield of ajmalicine (166 µg/g DW) was obtained from 20-d old cell cultures treated with *Trichoderma viride* [82]. In addition, the elicitation with a fungal preparation derived from *Penicilium citrinum* or PB90 (a protein elicitor from *Phytophthora boehmeriae*) also increased the production of catharanthine reaching levels over 20 mg/L in both cases in *C. roseus* cell cultures [83,111] (Table 1).

Another class of compounds that are gaining attention as potential elicitors of secondary metabolism in plants are cyclodextrins (CDs) which are modified cyclic oligosaccharides derived from starch. CDs have been able to induce defence responses in *C. roseus* cell cultures enhancing ajmalicine (over 200 mg/L) and catharanthine (85 mg/L) levels [73,80] (Table 1). 

Abiotic elicitors including UV-light or heavy metals also induce TIA production in *C. roseus*. Thus, hairy roots exposed to UV-B increased significantly the levels of lochnericine (over 2.7 mg/g DW), serpentine (over 0.62 mg/g DW), and ajmalicine (over 0.31 mg/g DW) [114]. Ramani and Jayabaskaran [84] also observed an enhancement of catharanthine (0.12 mg/g DW) and vindoline (0.06 mg/g DW) when *C. roseus* cell cultures were exposed to UV-B light. Moreover, a short UV-C irradiation promoted the production of 90 mg/L ajmalicine and 50 mg/L catharanthine in *C. roseus* cell cultures [73] (Table 1). 

In relation to heavy metals, the treatment of 50 µM chromium raised vincristine and vinblastine content in *C. roseus* plants, particularly in shoots reaching levels of around 2 and 2.25 µg/g DW, respectively [35] (Table 1). 

Biotic and abiotic elicitors are associated to different mechanisms of elicitation and, when used in combination, could enhance metabolite accumulation in plant cell cultures. Indeed, the joint action of *Aspergillus niger* mycelium and tetramethylammonium bromide induced a synergism on TIA accumulation in *C. roseus* cell cultures, since they increased the levels of ajmalicine (63 mg/L) and catharanthine (17 mg/L). Also, the combination of malate and sodium alginate resulted in a high ajmalicine (41 mg/L) and catharanthine (26 mg/L) accumulation [65,68] (Table 1). 

A new strategy for increasing TIA production in *C. roseus* cell cultures is the combined use of CDs and MeJA. In fact, when *C. roseus* cell cultures were elicited with both elicitors a high accumulation of ajmalicine and catharanthine was detected (450 and 155 mg/L, respectively). This effect was even greater when these elicitors were combined with short UV light exposure reaching a production of ajmalicine and catharanthine of 1040 mg/L (85 mg/g DW) and 196 mg/L (10 mg/g DW), respectively [73] (Table 1). 

## 5. Rational Approaches to the Biotechnological Production of TIAs

**Figure 2:**
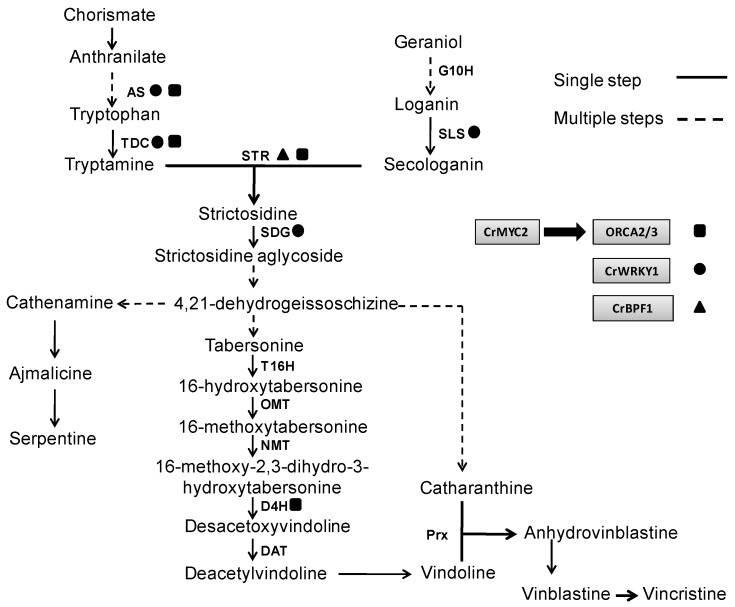
Gene regulation scheme of transcription factors involved in the TIA biosynthethic pathways. AS, anthranilate synthase; TDC, tryptophan decarboxylase; G10H, geraniol 10-hydroxilase; SLS, secologanin synthase; STR, strictosidine synthase; SGD, strictosidine β-D-glucosidase, T16H, tabersonine 16-hydroxylase, OMT, 16-hydoxytabersonine 16-O-methyltransferase; NMT, N-methyltransferase; D4H, desacetoxyvindoline 4-hydroxylase; DAT, deacetylvindoline 4-O-acetyltransferase; Prx, peroxidase.

If progress in the biotechnological production of TIAs is to continue, a rational approach to the molecular bioprocesses that take place in the producer cells is essential. In other words, it is necessary to know how the different empirical factors that increase yields of TIAs affect gene expression and metabolic profiles in *C. roseus in vitro* cultures. Such an approach can provide insight into biosynthetic pathways and their regulation. In this sense, the biosynthesis of TIAs involves a large number of enzymes which in turn, are highly regulated, and are located in different subcellular compartments acting in an organ and tissue-specific manner [4]. Precisely this compartmentalization and localization makes the TIA biosynthesis is considered a process tightly regulated, in which the different metabolic intermediates needs to be transported from one point to another for their transformation. TDC is found in the cytosol and G10H has membrane localization in endoplasmic reticulum as well as SLS which is anchored to the cytosolic face of the endoplasmic reticulum (Figure 2). As STR has been localized in the vacuole, secologanin and tryptamine can be translocated to this compartment to form strictosidine. This latter compound is transported out of the vacuole to form the strictosidine aglycone by SGD [115]. The next steps of the TIA pathway for the biosynthesis of tabersonine and catharanthine are quite poorly characterized, and nothing is known about their subcellular localization. As occur with G10H and SLS, the cytochrome P450, T16H are located in membranes, specifically in endoplasmic reticulum and the reactions catalyzed by the enzymes OMT, D4H and DAT to form vindoline are carried out in the cytoplasm, while the transformation to deacetoxyvindoline by NMT occur in the thylakoids (Figure 2) [116]. Ultimately, vindoline and catharanthine are transported to the vacuole, where a peroxidase (Prx) catalyzes their coupling to produce anhydrovinblastine, the precursor of vinblastine [117]. 

### 5.1. Elicitor Effects on the Expression of Genes Involved in TIA Metabolism

As mentioned above, empirical studies on *C. roseus in vitro* cultures have shown that TIA biosynthesis can be markedly induced by elicitors, however how the elicitors affect these specific metabolic pathways is not truly known [90]. In this way, different authors have studied the relationship between TIA accumulation and gene expression of *G10H*, *SLS*, *TDC* and *STR* under elicitation conditions, specifically in treatments with MeJA [80,118,119,120,121], CDs [80], and fungal homogenates [83,122,123] alone or in combination [80]. In fact, the transcript levels of *TDC* and *STR* were induced by MeJA treatments [80,119,120,121]. In contrast to these results, the expression levels of *SLS* and *G10H* were not increased [80] or increased slightly at short periods of time analyzed and then, these transcripts levels returned to the control levels at long treatment times [118,119,120]. This could be due to the fact that the expression profile of transcripts induced by MeJA in *C. roseus* depends on factors such as the developmental stage and type of the plant material, the cultivar of *C. roseus* and MeJA concentration used in the experiments. It is also important to note that the expression levels of these transcripts were always well-correlated with the levels of TIAs accumulated in MeJA-treated cells [80]. 

The expression of genes encoding enzymes from TIA biosynthesis can also be stimulated by adding biotic elicitors such as fungal preparations. Pasquali *et al.* [121] and Menke *et al.* [90] observed that *TDC* and *STR* genes in *C. roseus* cell cultures were strongly up-regulated after treatment with a filtrate of *Pythium* *aphanidermatum* and yeast extract. Similarly, Chen *et al.* [83] also demonstrated that *C. roseus* cells treated with PB90, displayed a time-dependent increase in catharanthine production involving a high level expression of *TDC* and *STR*. Similarly, CDs which have oligosaccharide structure and mimick fungal attack acting as elicitors [123], activated the expression of *G10H*, *SLS*, *TDC* and *STR* at all time points analyzed [80]. 

On the other hand, the combined effect of elicitors on the production of TIAs is a widely used strategy to increase TIAs, however there is few studies related with the joint action of elicitors on the expression of genes codifying enzymes from TIA biosynthesis. Almagro *et al.* [80] showed that the joint action of CDs and MeJA induced in *C. roseus* cell cultures, the highest expression levels of *G10H*, *SLS*, *TDC* and *STR*, observing a synergistic effect on both gene expression and TIA production. Therefore, it is evident that CDs activated a distinct pathway or played a different role in regulating TIA metabolism of that seen with MeJA in *C. roseus* cell cultures. Also the joint use of CDs and MeJA induced an important synergistic reprogramming of gene expression in *C. roseus* suspension cultured cells of *C. roseus*, which is probably the reason for the high accumulation levels of ajmalicine and catharanthine in those conditions. The molecular mechanism underlying the synergy between these two elicitors needs to be explored further.

### 5.2. Metabolic Engineering to Improve the Production of TIAs

Although there are only a few reports about the genetic transformation of *C. roseus*, metabolic engineering may be a potent tool for increasing TIA production in *C. roseus* cell platforms. Usually, enzymes of the biosynthetic pathway are selected as targets for gene cloning and then, manipulated by genetic engineering e.g., to raise metabolic flow rate toward the compound of interest and hence, enhance its levels. This approach has a limited value since a more predictable control of metabolic flux could be achieved using transcription factors because they can regulate one or more catalytic steps from TIA biosynthesis [124].

One of the major limitations to metabolic engineering of these systems is the lack of fully elucidated plant biosynthetic pathways. Although the “omics” era has led to an enormous increase in the available data, correct annotation and functional characterization of enzymes is crucial to the successful implementation of metabolic engineering strategies. In addition, the complex interactions amongst biosynthetic pathways and sophisticated regulatory mechanisms further complicate engineering efforts [125]. Therefore, amongst the strategies used to enhance TIA biosynthesis in *C. roseus in vitro* cultures, could be highlighted the overexpression of transcription factors and genes of their biosynthetic pathways as well as the overexpression of genes from the TIA biosynthesis in other organisms.

#### 5.2.1. Overexpression of Genes which Regulate TIA Biosynthesis 

Some studies were focussed on cell and tissue genetic engineering in *C. roseus*, resulting in higher yields of some TIAs [6,126] being *TDC* and *STR* the most overexpressed genes. In fact, *STR* overexpression in a transgenic *C. roseus* culture resulted in a significant increase of TIAs, reaching levels of 123 mg/L [86] (Table 1). Moreover, loganin feeding experiments provoked an increase in total TIAs of 300 mg/L [86]. Similarly, *TDC* overexpression did not increase [87] or slightly enhanced total TIAs levels (75 mg/L, [86]). However, when the precursors loganin and secologanin, were added to cell lines overexpressing *TDC*, the production of serpentine, catharanthine and strictosidine increased until 125-fold, resulting in a total TIA production of 625 mg/L [87] (Table 1). 

In a similar way, *C. roseus* hairy root cultures overexpressing *DAT* genes accumulated hörhammericine until 4-fold more than the wild-type (Table 1, [88]) while transgenic hairy roots overexpressing the gene encoding a Prx, accumulated 5- and 3-fold more serpentine and ajmalicine than their respective controls (Table 1, [89]). Moreover, as a result of overexpressing *geranyl diphosphate synthase* (gene encoding an enzyme that catalyzes the first reaction of terpenoid pathway) in *C. roseus* leaves, an increase in vindoline (Table 1) was observed [91]. Other study focused on the co-overexpression of several key genes including those monoterpenoid and indole pathway, as well as *G10H*, alone or in combination in hairy root cultures of *C. roseus* [115], resulted in a significant increase in ajmalicine, lochnericine, and tabersonine (Table 1). This experiment pointed out the need for overexpressing multiple genes within the TIA pathway to obtain significant results.

On the other hand, two genes involved in early steps of TIA biosynthethic pathway, *TDC* and *STR*, have been introduced in other cell cultures, specifically tobacco and *M. citrifolia* [92]. In these conditions, an increase of strictosidine was observed in both tobacco and *M. citrifolia* cell cultures fed with tryptamine and secologanin reaching levels of 21.2 and 5.3 mg/L, respectively (Table 1). Similarly, these two genes were overexpressed in *C. officinalis* hairy root cultures resulting in high levels of strictosidine (1.95 mg/g FW) [93]. Another strategy is the bioproduction of TIA using microorganisms like *S. cerevisiae* and *Escherichia coli* [94]. Thus, transgenic yeasts overexpressing *STR* and *SGD* were able to produce a high amount of strictosidine (2000 mg/L) when these cultures were fed with tryptamine and secologanin (Table 1) while a transgenic *E. coli* overexpressing *STR* was able to transform strictosidine in vallesiachotamine and isovallesiachotamine, two TIAs found in plants which belong to the family Apocynaceae [127]. 

#### 5.2.2. Overexpression of Transcription Factors which Regulate TIA Biosynthesis 

Most of genes codifying enzymes from TIA biosynthetic pathway are tightly regulated by specific transcription factors in a coordinate manner together with developmental and environmental factors. In *C. roseus*, only a few transcription factors have been isolated and characterized, highlighting the Octadecanoid-Responsive Catharanthus AP2/ERF-domain proteins (*ORCA2* and *ORCA3*), *CrBPF1*, *CrWRKY1*, *CrMYC1* and *CrMYC2* [73,78,79,80,81,82,83,84,86,87,88,89,90,91,92,93,94,95,112,113,114,115,116,117,118,119,120,121,122,123,124,125,126,127]. These transcription factors respond to jasmonic acid, MeJA, and/or elicitors. Two bHLH (a basic helix-loop-helix) transcription factors, *CrMYC1* and *CrMYC2*, were isolated and characterized from *C. roseus*. Both *CrMYC1* and *CrMYC2* were involved in the response to MeJA while *CrMYC1* also responded to fungal elicitors [128,129], suggesting that these transcription factors may be involved in the regulation of TIA biosynthesis in response to these signals. Moreover, *CrMYC2* activate the expression of two transcription factors, *ORCA2* and *ORCA3*, which bind to the JERE (a *cis*-acting element involved in jasmonic acid and elicitor responses) element of the *STR* promoter. However, while *ORCA2* respond to MeJA and elicitors, *ORCA3* only respond to jasmonic acid. Likewise, the isolation of *CrBPF1* transcription factor which is induced by elicitors but not by MeJA, suggest that elicitors induce *STR* gene expression via jasmonic acid-dependent and independent pathways [130]. 

On the other hand, *ORCA3* overexpression resulted in increasing levels of some genes involved in TIA biosynthetic pathway, specially *TDC*, *STR* and *D4H* [118,130] which in turn, provokes the accumulation of TIAs [97,99,131]. In fact, the overexpression of *ORCA3* in hairy root cultures increased the content of catharanthine up to 2.5-fold in comparison with the control cultures [97] (Table 1). Moreover, *ORCA2* is a transcription factor key in regulating genes encoding the enzymes *STR*, *TDC* and *SGD* involved in the first two branches of TIA biosynthetic pathway [98,132]. Liu *et al.* [98] also observed that the overexpression of *ORCA2* in *C. roseus* hairy root cultures provoked an increase of 2.0-fold in catharanthine (Table 1). Likewise, the combined overexpression of *G10H* and *ORCA3* in hairy roots provoked an increase of catharanthine (6.5-fold) [131]. The same overexpression was carried out in plants of *C. roseus* resulting in an important increase of vindoline, catharanthine, ajmalicine, anhydrovinblastine and vinblastine (Table 1, [99]). 

Other transcription factors involved in the biosynthesis of TIAs is the *WRKY* family. Overexpression of *CrWRKY1* in hairy roots increased the expression of genes encoding *AS*, *SLS*, *SDG* and *TDC* and repressed *ORCA2*, *ORCA3*, and *CrMYC2* resulting in an increase of ajmalicine and serpentine (Table 1, [95]). 

Finally, CrMPK3 is also an important component of intracellular signalling pathway triggered by different stresses in *C. roseus*. In fact, the overexpression of CrMPK3 in *C. roseus* leaves raised both the expression of *ORCA3* and genes involved in TIA biosynthetic pathway as well as serpentine, vindoline, catharanthine and vincristine levels (Table 1, [96]).

## 6. Conclusions

*C. roseus* is an amazing chemical factory, producing more than 130 TIAs, some of which exhibit strong pharmacological activities. The most striking biological activity investigated during recent years has been the antitumour effect of the dimeric alkaloids vinblastine and vincristine, as well as anhydrovinblastine, together with a number of semi-synthetic derivatives, known as the Vinca alkaloids. However, the scarcity of antitumoral TIAs found in *C. roseus* plants has stimulated an intense research effort aiming to obtain *C. roseus in vitro* cultures with higher production of these antitumour TIAs. To increase the production of TIAs, empirical strategies have been developed using plants, shoot, hairy root and cell *in vitro* cultures. Special attention has also been focused on experimental designs enhancing TIA production, which are the most promising strategies to improve TIA production in the future.

Metabolic engineering may be a potent tool for increasing TIA production in *C. roseus* platforms. Usually, enzymes of the biosynthetic pathway are selected as targets for gene cloning and then, manipulated by genetic engineering to raise metabolic flow rate toward the compound of interest and hence enhance its level. Indeed, the overexpression of genes encoding different transcription factors alone or in combination with key enzymes from the metabolic pathway especially in cells and hairy root cultures as well as in plants resulted in the developing of new improved biotechnological systems for producing important dimeric indole alkaloids such as anhydrovinblastine, vinblastine and vincristine, as well as the monoterpenic indole alkaloids vindoline, serpentine and lochnericine. A clear relationship is observed between the expression profile of the genes and the production of TIA.

The use of elicitors to activate genes involved in TIA metabolic pathways is an effective strategy to increase the biotechnological production of these compounds. Supplementing the medium with elicitors such as MeJA and CDs, individually or even in combination with UV light, induces an important reprogramming of gene expression in *C. roseus* cell cultures, which likely accounts for the observed enhanced production of some TIAs, specially tabersonine, ajmaline, ajmalicine and catharanthine. Although our knowledge increases, various aspects on the molecular mechanisms behind the action of elicitors and the possible synergy amongst them remain unknown. The ability of some elicitors like CDs to excrete these compounds to the culture medium is crucial for their biotechnological production. The use of high-producing *C. roseus* cultures elicited with CDs individually or in combination with other elicitors that excrete the bioactive compounds into the culture medium would allow the establishment of continuous systems in bioreactors without destroying the cell biomass, since the processes required for extracting and purifying of the target compounds would be simpler, more sustainable and economically more viable.
